# A Comprehensive Review of Optical Stretcher for Cell Mechanical Characterization at Single-Cell Level

**DOI:** 10.3390/mi7050090

**Published:** 2016-05-13

**Authors:** Tie Yang, Francesca Bragheri, Paolo Minzioni

**Affiliations:** 1 Department of Electrical, Computer, and Biomedical Engineering, Università di Pavia, Via Ferrata 5A, Pavia 27100, Italy; yangtie@gmail.com; 2 Institute of Photonics and Nanotechnology, CNR & Department of Physics, Politecnico di Milano, Piazza Leonardo da Vinci 32, Milano 20133, Italy; francesca.bragheri@ifn.cnr.it

**Keywords:** single-cell analysis, microfluidics, optofluidics, optical stretcher, mechanical properties characterization

## Abstract

This paper presents a comprehensive review of the development of the optical stretcher, a powerful optofluidic device for single cell mechanical study by using optical force induced cell stretching. The different techniques and the different materials for the fabrication of the optical stretcher are first summarized. A short description of the optical-stretching mechanism is then given, highlighting the optical force calculation and the cell optical deformability characterization. Subsequently, the implementations of the optical stretcher in various cell-mechanics studies are shown on different types of cells. Afterwards, two new advancements on optical stretcher applications are also introduced: the active cell sorting based on cell mechanical characterization and the temperature effect on cell stretching measurement from laser-induced heating. Two examples of new functionalities developed with the optical stretcher are also included. Finally, the current major limitation and the future development possibilities are discussed.

## 1. Introduction

During the last decades, the technologies that were initially developed and carefully optimized for microelectronic device fabrications widely expanded into other scientific research fields. One of the most relevant results of this “contamination” between microelectronic fabrication technologies and other research fileds led to the creation and development of the first microfluidic devices and lab-on-chip (LoC) systems. After their appearance in the 1990s, LoC systems have grown rapidly to become a very hot topic, due to the inherent advantages they offer with respect to “standard approaches”, including miniaturization, parallelization, integration, automation, as well as low consumption, high efficiency, rapid analysis, cost-effectiveness. Compared with conventional methods, LoC devices offer a great potential and they enabled new biomedical applications, ranging from drug discovery and delivery, to disease diagnosis and point of care (POC) devices. Since the size-scale of LoC internal structures is of the same order of most cells’ size, these devices have been extensively used for cellular biology studies, and in particular to analyze the biophysics and biomechanics of single cells [1,2,3,4].

Cell mechanical properties are mainly determined by the cellular cytoskeleton, which is a complex network of filaments, microtubules and linkers. It has been proved by many researches that cell mechanical properties are directly related to the cell status [5,6,7], like cell proliferation, differentiation and pathology transformation, particularly related to cancer. As an example, several studies demonstrated that cellular neoplastic and malignant transformations are closely connected with significant changes in the cytoskeleton, which are in turn related to changes in the mechanical properties of the cell [8,9,10].

Many different methods and experimental techniques have currently been proposed to assess cellular mechanical properties, either in a quantitative or qualitative way. For example, Vaziri, Pachenari *et al.* [11,12] applied a negative pressure in the micropipette to create an “aspiration region” on the cell and studied the local membrane deformation at the contact area; Mathur, Mackay, Rouven Brückner *et al.* [13,14,15] determined the local cellular Young’s modulus or the cell plasma membrane tension by using an AFM cantilever tip on the cells’ surface and measuring the relative indentation depth at constant force; Dao *et al.* [16] and Chen *et al.* [17] exploited optical tweezers or magnetic tweezers, with microbeads attached to the cell membrane, to apply a very large force onto the cell surface, and they derived the cellular viscoelastic moduli from the cell deformation. Preira, Luo, Martinez Vazquez *et al.* [18,19,20] developed a microfluidic chips with small constriction channels and applied them to the analysis of cell migratory capabilities, allowing to study both active and passive cell mechanical properties. However, some of these techniques can only access and hence probe a small portion of the cell, and most of them need a direct physical-contact between the studied cell and the device, which could modify cell’s natural behavior and even damage it during the measurement. Furthermore, these techniques often require quite complicated experimental preparations and they offer a relatively limited throughput. Recently, Otto, Mietke *et al.* [21,22] developed a purely hydrodynamic cell-stretching technique that allows increasing significantly the measurement throughput; this method is ideally suited when large populations of cells are analyzed, but it doesn’t allow cell recovery for further studies.

In contrast, the optical stretcher (OS in the following) proposed by Guck *et al.* [8] proved to be a very powerful tool for the study of cell mechanics: it is an optofluidic device combining the use of a microfluidic channel together with laser beams for optical stretching. The laser radiation applies a contact-less force on cell surface, causing a deformation that depends on cell mechanical properties. The use of a microfluidic integrated configuration allows achieving a high trapping (and analysis) efficiency of the cells flowing in the channel. Several studies already demonstrated that cell optical deformation measured from optical stretcher can be used as a mechanical marker to distinguish healthy, tumorigenic and metastatic cells, as well as to reveal the effects of drug treatments on the mechanical response of the cell [8,23,24,25].

In this paper we give a comprehensive review of the OS, including different fabrication techniques and materials, working mechanism and different applications. In addition, several new developments and findings from recent studies are also described.

## 2. Different Fabrication Techniques and Material

Thanks to the great improvement of micromachining technology, LoC and microfluidic device performance significantly advanced during the last decade. In this section we review the different materials and techniques that were reported in the literature for OS fabrication.

### 2.1. Basic Structure of an OS

**Figure 1:**
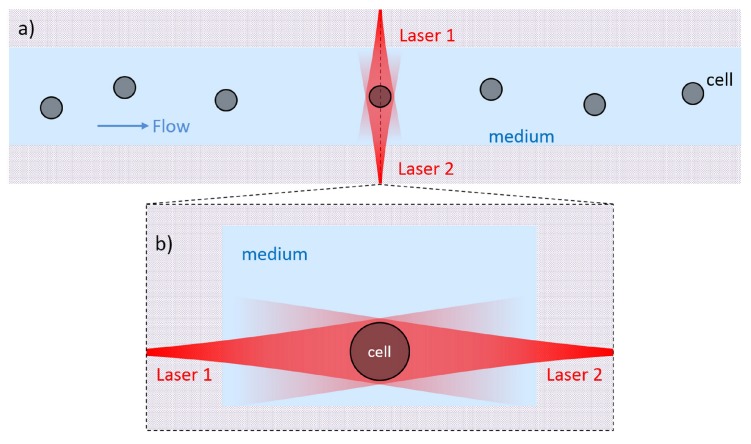
Schematic structure of an optical stretcher. (**a**) Top view of the central fluidic channel with flowing cells and a single cell trapped in the middle of the two opposing laser beams; (**b**) Cross section of the trapping area as indicated by the dash line in (**a**). The two opposite laser beams are positioned close to the channel floor.

The basic structure of an OS is schematically illustrated in Figure 1 and it is based on a dual-beam laser trap in a microfluidic circuit. The microfluidic network is typically composed by a single channel (even if multiple-input and multiple-output structures can be realized) allowing the cell suspension to flow from an external reservoir (e.g., a vial) to the laser trap and then to the output, which can be a sterile vial, or even a simple water drop. In order to achieve the best performance, the cross section of the channel should be rectangular, to avoid “lensing effects” from the channel-fluid interface, and the surface roughness should be extremely low, to allow a high imaging quality and to reduce the laser beam distortions at the interface. The laser trap should be designed and realized so that two identical counter-propagating beams cross the microchannel, generally in the “lower half” of the channel so as to easily intercept the cells flowing in the channel, e.g., 25 µm above the floor as reported in [26] , where cells with a typical dimension ranging from 5 to 20 µm are considered. The height of the flowing cells can be slightly modified by tuning the flow speed. It was experimentally found that a good height to position the optical trap is between 20 and 40 µm from the channel floor since it prevents the cells from depositing on the floor, while keeping the cells flowing slowly. Furthermore, the two laser beams should be preferably aligned perpendicularly to the flow direction, and they should be symmetrically positioned with respect to channel axis.

Different LoC systems and fabrication techniques were proposed in the literature to realize an OS, including semiconductors, polymers and glasses, each of them having specific properties and hence allowing to integrate different features in the final device [27]. As an example silicon allows for surface stability and thermal conductivity, but it is opaque, hence undesirable for imaging purposes. Polymers, which can be biocompatible and transparent, offer the advantages of low cost and availability of simple technologies for microchannel fabrication, even if their hydrophobicity and softness may be a problem for some applications. Glass, on the other hand has the advantage of being chemically inert, stable in time, hydrophilic, nonporous and it easily supports electro-osmotic flow. Moreover, when fused silica is considered, it possesses a very high optical transparency range, down to the UV, and very low background fluorescence, surface coating can be easily performed and optical waveguides can be integrated in the substrate.In the following, we summarize the different methods for OS fabrication.

### 2.2. Conventional Discrete-Elements OS

**Figure 2:**
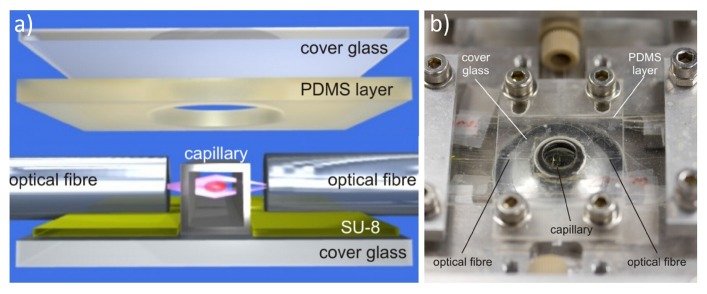
The structure of the discrete-element optical stretcher. (**a**) 3D rendering of the constituent components and their positions; (**b**) The finished system is mounted on a microscope plate. Figure reproduced from Reference [29] with permission from Optical Society of America.

Similarly to many optofluidic devices, the OS in its first implementation [8,28] was realized using discrete optical and fluidic components: two optical fibers were simply faced to a flow chamber where the cell suspension was flown. These first prototypes suffered from vibrations and mechanical drifts, which affect the system alignment and lead to barely repeatable experiments. To solve these problems, a solution reported in the literature is to align the optical and fluidic components on a substrate, by exploiting lithographically fabricated grooves, and then to seal the system with a suitable cover to increase the device robustness [26]. The fabrication procedure of such assembled optical stretcher (AOS) is illustrated in Figure 2a. A glass substrate is patterned with an SU-8 photoresist structure using standard photolithographic techniques. This leads to a single rectangular region of constant height (typically 35 µm) having perpendicular gaps to align and hold the optical and fluidic components. In particular a square glass capillary is used to transport the cell suspension and two optical fibers, single mode at wavelengths >1 µm (Hi-1060, Corning, New York, NY, USA), are used to create the dual-beam optical trap. A thin slab of polydimethylsiloxane (PDMS) with a 1.5 mm hole is placed over the setup so that the trap region is centered within the hole. The hole is filled with index matching gel to reduce reflection of the laser beams. A glass coverslip is secured over the PDMS piece. Finally the PDMS layer is screwed into the microscope stage and the capillary is connected to the external tubing network. In Figure 2b, the finished system is placed over an inverted phase contrast microscope for cell imaging.

### 2.3. Second-Generation Assembled OS

**Figure 3:**
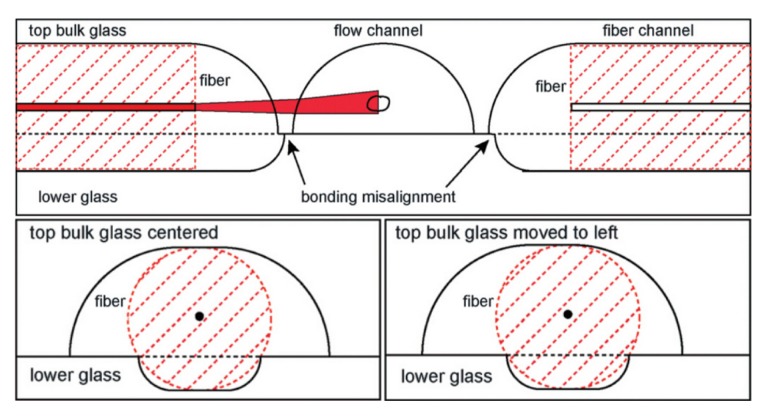
The chip geometry of the double glass layer assembled optical stretcher. The two glass layer are etched asymmetrically, the top one for large part of the fiber and the entire fluidic channel, the bottom one with shallow grooves for fiber alignment. The misalignment between these two layers show the robustness of this new etching layout. Figure reproduced from Reference [25] under CC-BY 3.0 license.

Two new methods to improve the tolerances of the AOS fabrication have been recently proposed. The first one is based on the use of two asymmetrically-etched glass substrates to accommodate both the flow channel and the fibers [25], as shown in Figure 3. The volumes etched from the top glass-layer include the majority of the optical fiber and the entire flow channel; on the other side, the bottom layer includes a shallow groove, for the fibers only. Through careful choice of the chip layout, good fiber alignment and cell trapping position can be achieved, together with a significant robustness to the misalignment of the two glass pieces. In fact, as shown in Figure 3, even a big misalignment of the top glass layer does not affect the flow channel and the fiber position. A small laser distortion is expected due to the etched curved surface, which can be minimized by fine tuning the geometry of the etching layout.

**Figure 4:**
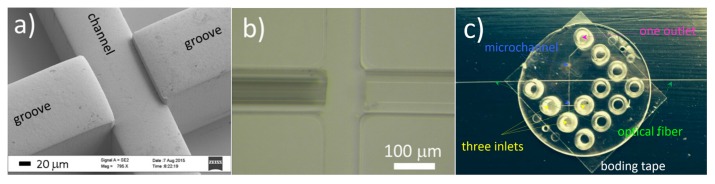
The polymeric assembled optical stretcher. (**a**) SEM image of the Ni shim at the area where the fiber grooves meet the channel. Higher position of the fiber grooves with respect to the central channel will lead to the lower position of the fibers after insertion; (**b**) Bright filed microscope image of the chip at the same area with a photonic crystal fiber (left) and single mode fiber (right) inserted; (**c**) The finished chip ready for use. The three inlets are for hydrodynamic focusing purpose. Figure reproduced from Reference [30] under CC-BY 4.0 license.

The second one is based on soft polymer material [30]. The proposed chip is fabricated by exploiting an innovative process, encompassing a double resist exposure and the use of Cyclin Olefin Copolymer (COC) TOPAS5013, which allows the fabrication of a multi-layer stamp shim. The two-level grooves for fiber positioning respect to the channel are obtained, as illustrated in Figure 4a. After the insertion of the optical fibers in the auto-aligning grooves they are glued and then the chip is sealed by thermal bonding of a TOPAS foil, see Figure 4b,c. An interesting feature of this approach is that different structure of the microfluidic channel can be easily obtained by changing the shim, and the easy chip fabrication method makes it ready for mass-production.

### 2.4. Femtosecond Laser Fabricated Monolithic Optical Stretcher

A completely different approach consists in the fabrication of a monolithic optical stretcher (MOS) by femtosecond laser micromachining (FLM) technology [29]. FLM holds many advantages over other fabrication techniques [31,32]: (i) it can be applied to different transparent materials; (ii) it enables rapid prototyping of devices; (iii) it is a 3D technique, allowing the fabrication of waveguides at different depths in the substrate; (iiii) it allows overcoming the complicated structure design and the assembly procedure. FLM has been extensively applied for a lot of optofluidic microchips in many studies. By the technique known as Femtosecond Laser Irradiation followed by Chemical Etching (FLICE) [33,34,35,36], the direct fabrication of microfluidic channels in fused silica can be obtained, which makes possible the integration of the microfluidic network and the optical waveguides by the laser radiation in the same substrate.

**Figure 5:**
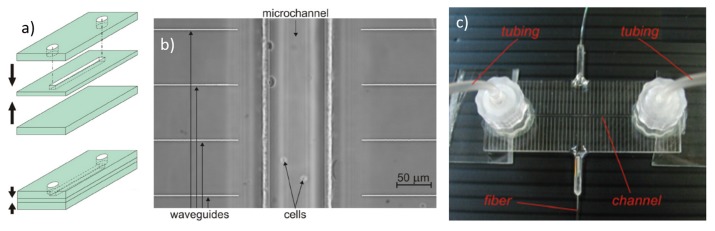
(**a**) Schematic representation of the three-layer technology for the monolithic optical stretcher fabrication. The central fused silica glass is machined by Femtosecond Laser Irradiation followed by Chemical Etching (FLICE) technique for the central channel and then sealed on both sides with two polished glass slides; (**b**) Microscope image of the straight microfluidic channel with pairs of waveguides beside it; (**c**) The finished chip is pigtailed with optical fibers and connected with external tubing through Luer connectors. Figure reproduced from Reference [29] with permission from Optical Society of America.

The first example of a MOS realized by FLM was obtained by integrating optical waveguides into a commercial microfluidic chip produced by Translume Inc. (Michigan, MI, USA). The chip is based on a “3-layers technology” where a fused silica glass slide with a thickness of 250 µm is machined with the FLICE technique to obtain a through slot as the fluidic channel [29,37], see Figure 5. The machined layer is then placed in the middle of the structure and sealed by thermal bonding with two polished fused silica glass slides on both top and bottom surfaces . In particular, while the bottom layer is a simple, unmodified, slide, the top layer has two through-holes aligned with the middle-layer channel terminations, so as to form the input and output accesses of the microchannel. The subsequent fabrication of pairs of opposing waveguides orthogonal to the channel was also realized by femtosecond laser writing, which allows adjusting the waveguides “depth” with respect to the channel during the laser writing process. With such a fabrication technology, the channel cross-section has a perfect rectangular shape with optical quality for the top and bottom channel walls and very low surface roughness of 200 nm rms for the lateral walls, thus allowing for a good imaging quality and a high efficiency optical-trapping by the dual beam configuration. Additionally the opposing waveguides are also aligned with a very high precision, thus allowing to obtain a robust, portable and highly flexible monolithic OS, see Figure 5c.

A different approach of monolithic OS, fully realized by FLICE in a single piece of silica glass, was demonstrated by Bellini *et al.* and Bragheri *et al.* [36,38]. Despite the significant advantages given by the possibility to realize in a single “writing procedure” both the microchannel and the optical waveguides, this method showed the significant drawback of producing microchannels with a high surface roughness, which can lead to a low image quality for the cell imaging. However, in a recent paper by Yang *et al.* [39] a new laser writing geometry allowing to decrease the surface roughness and to strongly improve the image quality was reported and further discussed in Section 5.1.

## 3. Working Principle of the OS

In this section we briefly review the basic physical principles underlying the mechanism of an OS, which is independently of its design, material and fabrication technique. In particular, we give a detailed description of the optical force distribution calculation, of the cell optical stretching procedure and of the method used to characterize cell deformation.

### 3.1. Optical Forces for Cell Stretching

As optical tweezers, also the OS exploits optical forces to both trap cells, but differently from tweezers, the scattering force in OS can be effectively used also to stretch the samples. Each photon from the laser beam carries a specific momentum, given by Equation (1), where *h* is the Planck constant; *λ* and *υ* the wavelength and frequency of the light; $c_{0}$ the light speed in vacuum; and *n* the refractive index of the traveling material. This momentum is modified both in modulus and direction when the photon crosses through the interface between the external medium and the cell. To correctly evaluate the force distribution applied on the cell surface it is important to take into account that both reflection and transmission occur at the interface between the cell and the suspension medium; thus a different momentum-change is experienced by reflected and transmitted photons. The ratio between reflected and transmitted photons is given by Fresnel equations, which are generally applied under the hypothesis of unpolarized radiation, and hence depends on the angle formed between the photons propagation direction and the cell surface, as well as by the refractive indices of the medium and the cell (with the general assumption that *n*${}_{cell}$ > *n*${}_{medium}$).

(1) $$\left.\begin{array}{c} p=h/\lambda \\ c=c_{0}/n=\lambda \upsilon \end{array}\right\}\;\;p=\frac{hn}{c_{0}\upsilon }$$

Since, up to now, no method allows a direct measurement of the optical force distribution on the cell surface, some mathematical and computational models have been proposed to calculate it. One of the main problems is to obtain a proper determination of the force applied by the impinging laser radiation on each point of the surface. Starting from the seminal works by Ashkin *et al.* [40,41,42], it is possible to evaluate the overall optical force applied on a dielectric sphere by an impinging optical beam thanks to its decomposition into a series of optical rays which are assumed to compose the optical beam (standard ray optics—SRO). This ray optics approach was then adapted by Guck *et al.* [8,28] for the optical force distribution on the cell surface inside the optical stretcher. And his model is valid because normally the studied biological cell samples have a much bigger size (~10 µm) than the wavelength of the applied laser light, which is called large particle regime. This approach is however not sufficient to describe the full laser-particle interaction when loosely-focused (or collimated) beams are considered, as in that case the interaction region generally lies within the beam Rayleigh-range, and thus a different beam-decomposition technique (paraxial ray optics—PRO, [43]) has to be applied. Afterwards, some other studies improved this method by including the effects of multiple internal reflections of the laser light inside the cell [44,45,46].

In the following we briefly review the description of the PRO approach, so as to give the reader a better understanding of the beam decomposition and optical force calculation technique. The optical force calculation is basically performed as a two steps process: first an adequate decomposition of the optical beam as a series of optical rays in the region of interest (*i.e.*, in the area occupied by the cell/particle) is calculated, and then the interaction of each optical ray with the cell/particle is calculated, thus allowing to evaluate also the overall beam effect on the sample.

**Figure 6:**
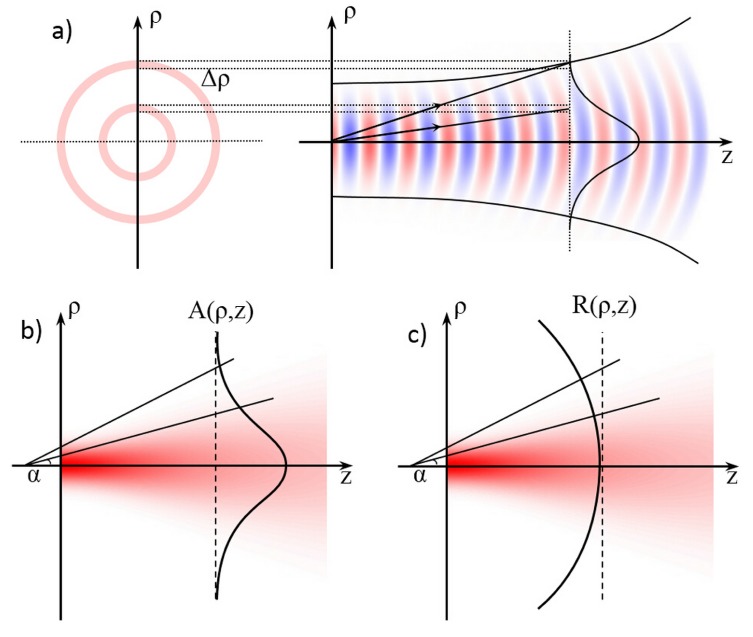
Scheme of the optical field determination from a Gaussian laser beam with the paraxial ray optics approach. (**a**) The power carried by each ray, is calculated as the integral of the beam intensity, as a function of the radial coordinate within the area of the annulus associated to the ray; (**b**) The amplitude *A*(*ρ*, *z*) and the (**c**) curvature radius *R*(*ρ*, *z*) along the axis *z* are calculated, which will be exploited respectively for the evaluation of the power and the propagation direction of each ray.

The first step, as shown an sketch in Figure 6a, is to decompose a non-focused Gaussian laser beam (as that generally emitted by optical fibers) into a distribution of individual rays, each characterized by a tproper direction and position in space, and carrying a certain amount of optical power. Considering the far field distribution through an angular spectrum decomposition technique, the power carried by a ray that intercepts the coordinate *z* at a distance $\rho_{0}$ from the axis of the beam is calculated as the integral of the intensity as a function of the radial coordinate *ρ*, in the portion of the annulus delimited by Δρ, see Figure 6a. The spatial phase gradient is then used to determine the propagation direction of each ray, which is perpendicular to the wavefronts. In particular, by adopting the paraxial approximation, and considering a Gaussian beam with waist $\omega_{0}$ at z=0, the optical field can be analytically described by two simple equations giving the electric-field amplitude (*A*), and radius of curvature (*R*) of the wavefronts respectively

(2) $$A\left(\rho ,z\right)=A_{0}\frac{\omega_{0}}{\omega_{z}}\exp\left(-\frac{\rho^{2}}{\omega_{z}^{2}}\right)$$

(3) $$R\left(z\right)=z+\frac{z_{R}^{2}}{z}$$

where $z_{R}$ (the so called Rayleigh range) and $\omega_{z}$ (beam width as a function of propagation distance) are:

(4) $$z_{R}=\frac{\pi \omega_{0}^{2}n}{\lambda }$$

(5) $$\omega_{z}=\omega_{0}\sqrt{1+{\left(\frac{z}{z_{R}}\right)}^{2}}$$

The amplitude of each ray is used to assess the optical power carried by each ray *P*, whereas the curvature radius is used to determine its propagation direction, thus obtaining a precise description of the beam properties in the area occupied by the cell/particle.

**Figure 7:**
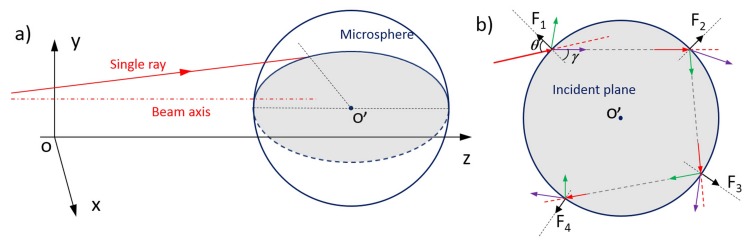
Coordinate system for a single ray interacting with a particle. (**a**) A single rays from a Gaussian laser beams hits on the surface of a particle. The locations of the laser beam and particle can be random. The incident plane is defined by the ray and the normal direction of the particle at the hitting point and is indicated by the gray area; (**b**) the single ray undergoes multiple reflections and refractions on the boundary of the particle.

Then, as a second step, the interaction of each single ray with the cell/particle is considered. The reference system used to evaluate the interaction between each ray and the particle is shown in Figure 7a. We assume for the calculations that the particle has refractive index ($n_{p}$) larger than that of the surrounding medium ($n_{m}$). According to the analysis already reported in the literature by Ashkin *et al.* and Brevik [42,47], it it possible to evaluate the force transferred by each single optical ray because of its interaction with the particle surface, due to the difference of the refractive indices, as it undergo multiple reflections and refraction on the boundary of the sphere, see Figure 7b.

Taking the first ray-particle interaction as an example, the incident angle is defined by the geometries of both the Gaussian laser beam and the particle and is referred as *θ*. According to Snell’s law and Fresnel equations, the refractive angle *γ* and the transmission coefficients (*T*) and reflection coefficients (*R*) can be calculated exactly. Then, the momentum carried by the photons in the incident, reflection and refraction beams can be calculated through Equation (1). We denote the momentum of the incident, transmitted and reflected rays by $\vec{p_{i}}$, $\vec{p_{t}}$ and $\vec{p_{r}}$, and their directional unit vectors by $\vec{a_{i}}$, $\vec{a_{t}}$ and $\vec{a_{r}}$ respectively. According to the momentum conservation law, the stress $\vec{\sigma }$ applied on the local surface of the particle is expressed as the following equation [44]:

(6) $$\begin{array}{cc} \vec{\sigma } & =\frac{\Delta \vec{p}}{A\Delta t}\\ & =\frac{\vec{p_{i}}-\left(\vec{p_{t}}+\vec{p_{r}}\right)}{A\Delta t}\\ & =\frac{n_{m}}{c_{0}}\frac{P}{A}\left[\vec{a_{i}}-\left(\frac{n_{s}}{n_{m}}T\vec{a_{t}}+R\vec{a_{r}}\right)\right]\\ & =\frac{n_{m}}{c_{0}}\frac{P}{A}\vec{Q} \end{array}$$

where *A* is the area of the particle irradiated by the single ray, *P* is its optical power, $c_{0}$ is still the light speed in vacuum and $\vec{Q}$ is defined as a dimensionless momentum transfer vector. Furthermore, it is proved that the direction of this optical stress is always perpendicular to its acting surface and pointed away from the optically denser part [8,44], as shown in Figure 7b. In the same way, the optical stress from the following interaction of this single ray can be calculated and this can be easily adapted to other single rays by only changing the first incident angle and the associated power. The sum of the optical force from each ray results in a optical force distribution on the surface of the particle.

**Figure 8:**
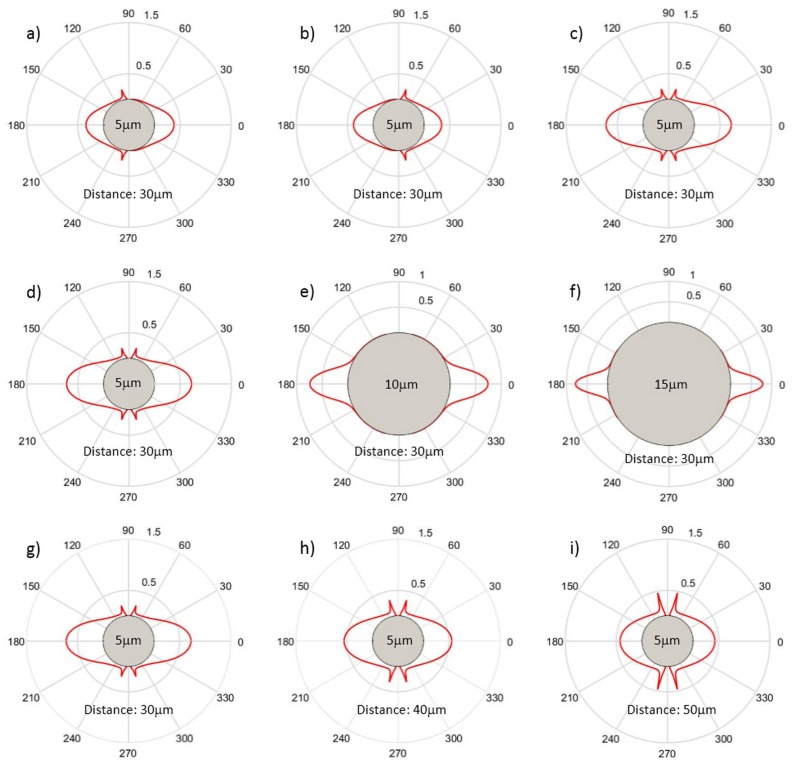
Polar plots of the calculated optical stress distribution on the surface of the particle. The Gaussian laser beam has beam waist of 3.1 µm and wavelength of 1.07 µm and carries optical power of 10 mW. The refractive index of the particle is 1.37 and medium 1.33. The distance between the particle center and the beam waist (either one laser or two lasers) is indicated in each panel together with the diameter of the particle. (**a**–**c**) show the optical stress from left side laser radiation, right side laser radiation and both side laser radiation respectively; From (**d**) to (**i**), the two-side laser irradiation is considered; (**d**–**f**) show the optical stress distribution change with particle size increase; (**g**–**i**) show the optical stress distribution change with distance increase.

As an example, Figure 8 shows the calculated optical force distribution on a particle under different conditions. The applied Gaussian laser beam has a wavelength of 1.07 µm, a beam waist of 3.1 µm and carries an optical power of 10 mW. The particle, which is considered to have a diameter of 5 µm and to lay 30 µm away from the beam waist position, exactly along the laser beam axis, has refractive index of 1.37, similarly to real cells, and the surrounding medium has refractive index of 1.33 (corresponding to that of water). The optical stress distribution produced on the particle surface by a single laser beam shining from left to right and from right to left is shown in Figure 8a,b respectively. The resulted optical stress profiles are rotationally symmetric with respect to the beam axis and there is net force from a single laser beam pushing the particle away from the laser source. It is interesting to notice that the “spikes” which are observed in the stress profile are produced by the second order internal laser-particle interaction [44], and they tend to become more relevant as the particle diameter becomes smaller with respect to the beam one. When the two counter-propagating beams are simultaneously impinging on the particle, see Figure 8c, the net applied force is zero, and the particle is stably trapped in the center. However, the local optical force acting on the surface is not zero and it significantly increases with respect to the single laser case [8]. This situation is that commonly exploited to create an OS, by simply increasing the laser beams power. In Figure 8d–f we show the optical stress distributions of different particle size (5 µm, 10 µm, 15 µm) under the same situation of Figure 8c. Figure 8d is the same as Figure 8c and it is shown only to help the comparison. By comparing the three panels of the second line, it can be immediately seen that as the particle size is increased the optical force becomes more concentrated along the laser beam axis, and the stress distribution-shape slightly changes. Figure 8g–i show the optical stress distribution on a 5 µm particle when the distance of the particle from the beams waist is set to 30, 40 and 50 µm, respectively. By increasing the distance, the optical stress distribution slightly changes, and the overall stress is decreased, as can be intuitively understood noticing that the overall intercepted power is reduced as the beam broadens during propagation because of diffraction.

### 3.2. Cell Stretching Procedure

**Figure 9:**
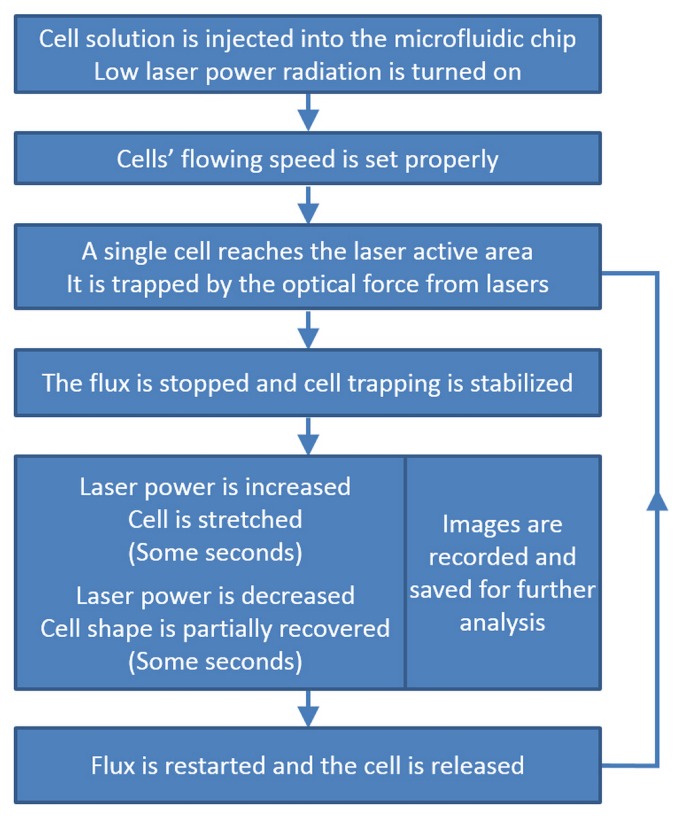
Flow chart of continuous cell stretching procedure.

Similar procedures for cell stretching measurements are reported in the literature [8,23,24,29] and the work-flow can be graphically depicted in Figure 9. First, the cell sample is injected by external pump system into the microfluidic circuit of the OS and low laser power is turned on for the two opposing laser beams. The cell flow is then adjusted as fast as possible, provided that the laminar flow is maintained and both a good imaging of flowing cells (which depends on the camera frame rate) and an efficient trapping (which depends on the cell velocity with respect to the laser power) are assured. Once a single cell reaches the laser irradiated zone and is trapped by the optical force, the flux is stopped, so that no additional cells reach the “trapping area”. After this, the optical power output by the two waveguides is increased to the preset “stretching power” value with a step-like power profile and the cell progressive deformation is recorded by a camera. After a certain time interval (typically about 5 s), the laser power is switched back to the low “trapping” value, and the stretched cell is observed while it partially recovers the original shape. During the stretching and recovery processes, the images are recorded and saved at a high frame rate, to allow for subsequent image analysis. Finally, the studied cell is released, by switching off the laser beams and restarting the flux, and the whole procedure is then repeated for other single cells (additional details can be found in [8,23,29]).

### 3.3. Characterization of Cell Optical Deformation

**Figure 10:**
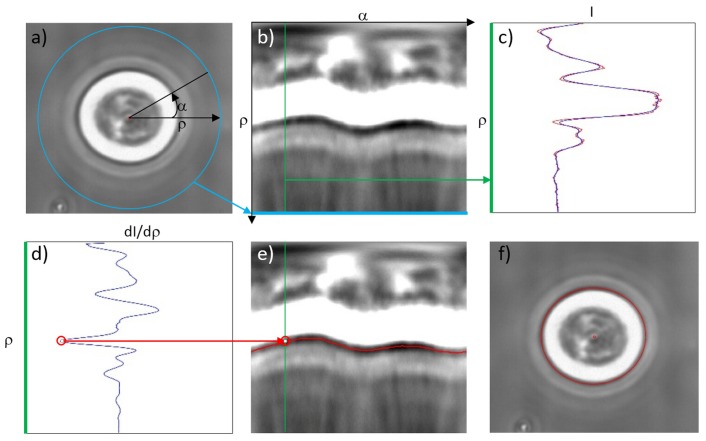
Illustration of the image analysis steps. (**a**) Phase contrast microscope image of a single cell. The center point (red color) of the cell is manually selected and the circular border (light blue color) for the polar transformation is determined by the original rectangular image border; (**b**) The polar-transformed image; (**c**) shows a plot of the gray-scale intensity along the green vertical line appearing in (**b**). The raw data (red line) is smoothed by Fourier filtering (blue line); (**d**) shows the first derivative of the blue-line (intensity) and the red circle shows the point identified as minimum, identified as belonging to cell border; (**e**) by repeating the same procedure for all the polar angles, the cell border is reconstructed on the polar image and (**f**) then transformed back to original image.

In order to characterize the cell optical deformation from stretching measurements, the previously stored images are analyzed. There are different methods reported in the literature [8,26,48]. As an example, here we show the method, exploited by Guck, Lincoln, *et al.* [8,26], based on the image polar transformation and edge detection algorithms. The procedure is schematically represented by the different steps shown in Figure 10. First of all, each image is transformed into a polar coordinates system, by exploiting an automatic technique for cell-center identification. The radius limit of the polar coordinate is determined by the original rectangular image border, see the blue circle in Figure 10a, which is also present in Figure 10b as a straight line after polar transformation. Accordingly, the bottom part of Figure 10b is the outside of the cell.

The intensity of the gray scale image for each angular direction (*i.e.*, each vertical line in panel (b)) is extracted (see Figure 10c showing the intensity profile corresponding to the green line in Figure 10b) and smoothed by a low-pass Fourier filter to remove the small spikes. Then, the derivative of the intensity values along the radius is calculated and the minimum point (corresponding to the fastest transition from bright to dark region around the cell border) is recorded, as indicated by the red circle in Figure 10d. For each angle, the same process is applied and the cell border at each polar angle is obtained, see Figure 10e. Then this cell border curve in polar coordinates is transformed back and the cell contour is plotted upon the original phase contrast image as shown in Figure 10f. For the edge detection, some other signal processing methods can be additionally implemented to optimize the detection result, like squaring the intensity profile to have a stronger contrast or defining a threshold to simplify the global minimum search as demonstrated by Guck *et al.* [8]. With this method, a very high resolution of 100 nm can be obtained, which can satisfy the purpose of cell border recognition.

**Figure 11:**
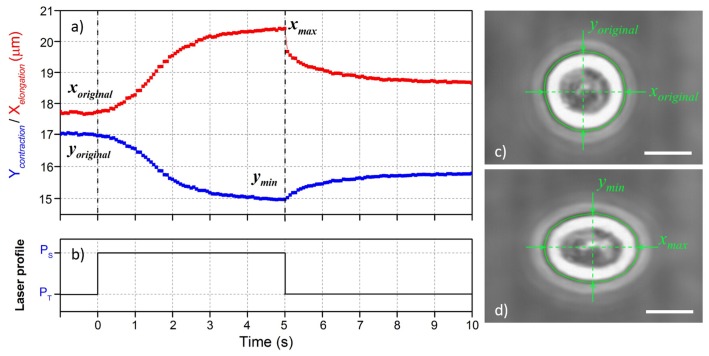
Single cell optical stretching. (**a**) Cell dimension variation during optical stretching together with the laser power profile (**b**): P${}_{T}$ is trapping laser power of 25 mW per side and P${}_{S}$ is stretching power of 1.5 W per side. *X*-axis is along the laser beam and *Y*-axis along the cell flowing direction; (**c**,**d**) show the phase contrast microscope images of the same cell trapped and stretched respectively. Green contours are cell borders identified by the recognition algorithm. Scale bars in both (**b**,**c**) are 10 µm. The cell sample is human breast cancer cell MCF7.

The image analysis procedure is applied to all the images from the cell optical stretching measurement and the cell contour is found in every image frame. Afterwards, the cell optical deformation in terms of elongation along the laser beam axis and contraction in the perpendicular direction can be derived from these cell contours. As an example, Figure 11 shows the deformation profile of a single cell (MCF7) under 5 s optical stretching and further 5 s recovery. For the stretching, a typical creep compliance curve of cell size variation can be observed. The cell deformation is reported as the absolute cell size value. The cell is increasingly elongated (*X*${}_{elongation}$) along the laser beam axis and contracted (*Y*${}_{contraction}$) in the perpendicular direction under stretching duration. After stretching, the cell can partially recover its shape. Figure 11c,d show the microscope images of the original cell trapped at the very beginning of the stretching and maximally stretched after 5 s of high-power irradiation.

To explain this time dependent and compliance behavior of the cell deformation in response to a constant step stress from the optical stretching measurement, Wottawah *et al.* [49,50] applied constitutive equations and, by fitting the experimental result with the theoretical prediction, the viscoelastic parameters of the cell, like Young’s modulus or shear modulus, can be derived. Some other studies proposed more complicated mechanical 3D models to mimic the real complex structure of a single cell and study the different contribution of cellular mechanical structure. Ananthakrishnan *et al.* [51,52] developed two structural models: a thick shell model for the actin cortex and a three-layered model for the whole cell, and they found that the outer actin cortex mainly determines the structural response of the cell during cell stretching. Another interesting result was obtained by Gladilin *et al.* [53], which created a three-component model (including the nucleus, the cortical actin filament and the perinuclear vimentin intermediate filament) allowing them to isolate the contribution of each component, thanks to the use of specific drug treatments.

Besides, the cell maximum relative deformation (*i.e.*, strain *ϵ*) is also often used in literature as a simple reference parameter for cell mechanical property comparison. It can be either the relative elongation (Equation (7)) or the relative eccentricity variation (Equation (8)). In both equations, there is a correction term “corr” obtained by numerical simulations, which accounts for the variations of the optically induced stress profile from different cell size and the refractive index of the different cells as described in reference [8,23].

(7) $$\epsilon \left(\%\right)=\left(x_{max}/x_{original}-1\right)\cdot 100\cdot corr$$

(8) $$\epsilon \left(\%\right)=\left(\frac{x_{max}}{y_{min}}/\frac{x_{original}}{y_{original}}-1\right)\cdot 100\cdot corr$$

## 4. OS as a Tool to Analyze Cell Lines, Drug Treatments and Cellular Organelles

Since its invention, the OS clearly demonstrated its ability to test cell mechanical properties, and during the last decade, it has also been extensively and successfully applied to study different cell samples, the effect of various drug treatments on cell mechanics and internal cellular organelles.

### 4.1. Optical Stretching of Red Blood Cells and Lipid Vesicles

**Figure 12:**
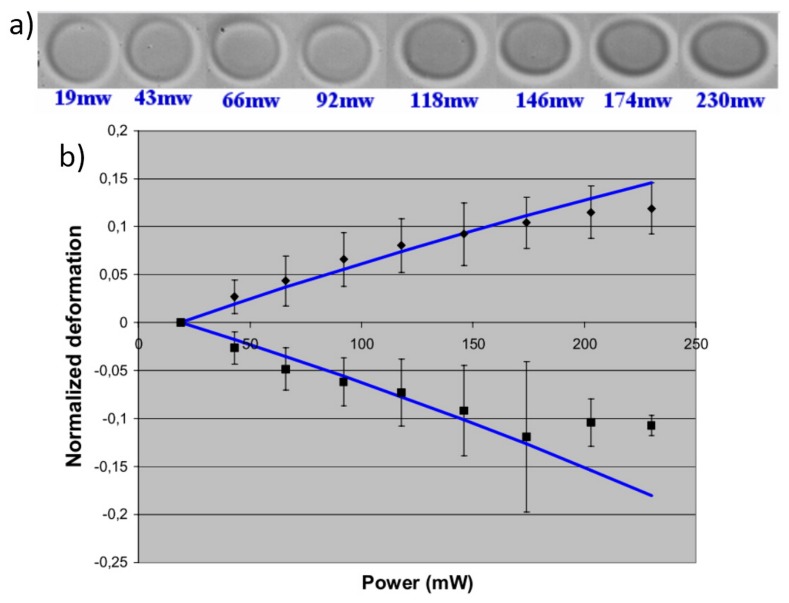
Optical stretching of single red blood cell (RBC). (**a**) Microscope images of RBCs stretched at increasing optical powers; (**b**) Optical deformation of RBCs in terms of elongation along laser axis and contraction in the perpendicular direction at different stretching power. The laser power is the total power for both laser beams. Experimental data is fitted with theoretical prediction from the linear elastic membrane theory. Figure reproduced from Reference [45] with permission from Optical Society of America.

The first application of the OS to the study of single cell mechanics is related to the study of red blood cells (RBCs) [8]. The simple structure of RBCs (they do not have any organelles or internal structure), and the possibility to make them almost perfectly spherical by swelling in a hypo-tonic suspensions makes them to be the ideal candidate for preliminary analysis, as they can be effectively modeled as homogeneous spheres, with an isotropic index of refraction, thus simplifying the analysis of cell mechanical response. In Figure 12a, it is shown the microscope images of single RBC stretched at gradually increased laser power levels [45]. The cell is elongated along the laser beam axis and contracted in the perpendicular direction. Given the optical field of the stretching laser (magnitude and direction), the RBC size and its position in the optical field, the refractive indices of the cell and the buffer medium, the optical force applied on the cell surface can be precisely evaluated, as discussed in Section 3.1. The RBCs’ relative deformation is represented in Figure 12b and it can be seen that the deformation of RBCs maintain a linear response with respect to the optical power until 150 mW. Within this linear regime, linear membrane theory can be used to describe the deformation of RBCs [8,45] (see the curve in the figure) and a value of Young’s modulus about $Eh=\left(20\pm 2\right)\mu Nm^{-1}$ was derived by Bareil *et al.*

Afterwards, many other related studies on optical stretching of red blood cells have been performed: Bareil, Ekpenyong *et al.* [44,45,46] calculated the exact stress distribution on the cell surface for a more accurate derivation of the cell stiffness; Ye, Mauritz, Sawetzki *et al.* [54,55,56] studied and compared the mechanical property of healthy *vs.* malaria-infected RBCs; Sraj *et al.* [57,58] applied laser diode-bar for RBCs optical stretching and proved a single-beam, high-throughput method for cell deformation cytometry; Liu , Tan *et al.* [59,60] developed a mechanical model and conducted a systematic simulation of RBCs dynamic deformation in optical stretching experiment.

**Figure 13:**
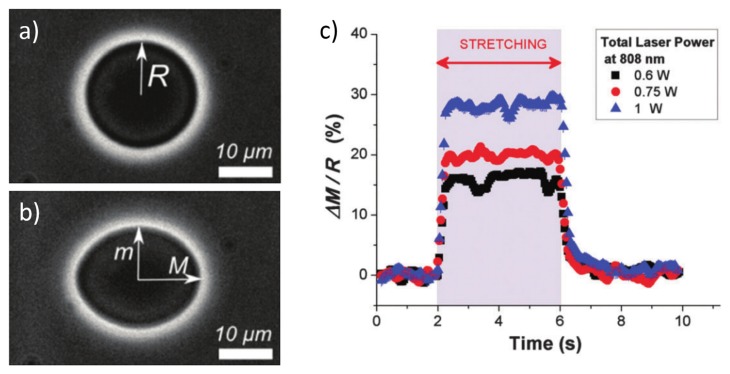
Optical stretching of vesicles. Microscope image of a vesicle trapped at low power (**a**) and deformed at high power (**b**); (**c**) The major axis strain of vesicles under 4 s stretching at various total powers. Figure reproduced from Reference [62] with permission from Royal Society of Chemistry.

Other studies showed the analysis of phospholipids vesicles (synthetic structures with a spheroidal lipid-bilaryer shape) by exploiting the OS measurement technique [50,61,62]. Similar to RBCs, they can provide a simple mechanical model for studying membrane mechanics, which is a very important aspect of the cellular function. In Figure 13, an example of a vesicle trapped at low laser power and then stretched at high power is reported. The time resolved deformation of vesicles under different laser power is plotted in Figure 13c. It is clear that vesicles have a fast response to the applied optical stimulus as they can reach their final deformation immediately after the stretching started and recover the deformation and return to the initial shape very rapidly after the stretching stopped. Interestingly, by stretching vesicle, Solmaz *et al.* [61] were able to extract the bending modulus of the lipid bilayer, while Delabre *et al.* [62] proposed a vescicle model based on a quasi-spherical approximation allowing also to take into account the laser heating effect on the vescicle deformation.

### 4.2. Optical Stretching of Eukaryotic Cells and Drug Treatment Effect

**Figure 14:**
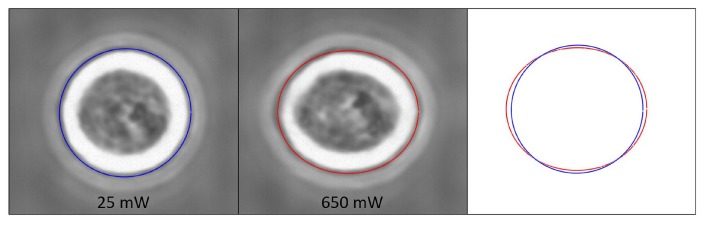
Optical stretching of a single MCF7 cell. The laser power is for each side and the cell contour is recognized by the edge detection algorithm in Figure 10.

Differently from erythrocytes, eukaryotic cells have a much more sophisticated structure including various internal organelles, nucleus and several immersed polymer networks. In particular, these polymer networks, which consist mainly of three different filamentous proteins: actin filaments, microtubules, and intermediate filaments, form the cellular cytoskeleton and provide the basic mechanical support for the whole cell in terms of mechanical strength and morphology [23,50]. Guck *et al.* [8] first exploited the optical stretcher to analyze BALB 3T3 fibroblast cells and showed that even if a very high laser power was applied, the cell showed a very small deformation. Because of its internal structure, BALB 3T3 cells behaved like a relatively hard sphere. Similar behaviours are obtained for different populations of eukaryotic cells. Figure 14 shows an example of a single MCF7 cell trapped at low power of 25 mW per side and stretched at much higher power of 650 mW per side; even in this case only a small deformation can be seen.

**Figure 15:**
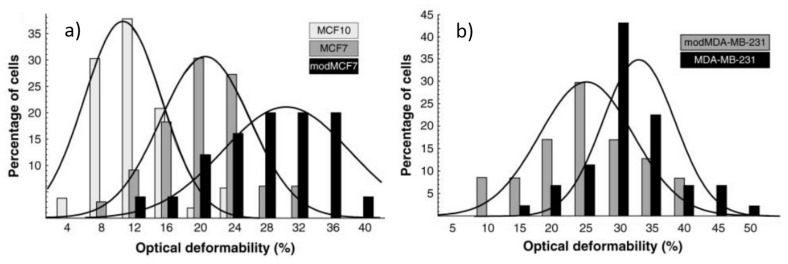
Optical deformability of normal, cancerous, and metastatic breast epithelial cells. (**a**) The three populations of the MCF cell and (**b**) the two populations of the MDA-MB-231 cell are clearly distinguishable. Curves represent the fitting of normal distribution. Figure reproduced with permission from Reference [23] with permission from Prof. Guck.

After the successful application of the optical stretcher to eukaryotic cells, Guck *et al.* [23,26] exploited it to evaluate variations in the mechanical properties of cell lines at different evolution stages, e.g., the healthy cells with respect to the tumorigenic ones and even the metastatic ones. The effects of drug treatments on cell mechanical property have been also investigated. In particular, a well characterized cell line of human breast epithelial cells and their cancerous counterparts were considered by Guck and coworkers: MCF10, MCF7 and MDA-MB-231, which are respectively normal, cancerous and highly metastatic cells. In Figure 15, the optical deformability of the three cell lines is reported. The results showed that the curves of the cells’ optical deformability can be fitted with a normal distribution and the obtained optical deformability values are: 10.5% ± 0.8% for MCF10, 21.4% ± 1.1% for MCF7 and 33.7% ± 1.4% for MDA-MB-231. With few cells measured, their optical deformability can be surprisingly distinguishable in a statistic way. The differences in the optical deformability of the three cell lines can be directly related with their different metastatic potentials.

Besides, they applied trug treatments to the two cell lines of MCF7 and MDA-MB-231 and their results are also included in Figure 15. For MCF7 cell sample, the phorbol ester TPA was applied, which can dramatically increase MCF7 cell invasiveness and its metastatic potential; it can be observed in Figure 15a that the optical deformability is increased with this drug treatment (modMCF7) highlighting higher optical deformability corresponds to the higher metastatic potential. While, MDA-MB-231 cells treated with alltrans retinoic acid, became less aggressive and its optical deformability was decreased, see (modMDA-MB-231) in Figure 15b, which showed the reverse trend of metastatic competence with cell optical deformability.

Other cell lines have been also evaluated, Lautenschläger *et al.* [63] studied acute promyelocytic leukemia (APL) cells with optical stretcher and revealed a significant softening during differentiation. Furthermore, they exposed the cell to paclitaxel and found out that this treatment does not alter cells’ compliance from optical stretching but reduces cell relaxation after the optical stress is removed. Schulze *et al.* [64] exploited the optical stretcher on human skin fibroblast cells and showed that an increase in age was clearly accompanied by a cell stiffening. Ekpenyong *et al.* [9] evaluated the differences in cell mechanical properties during differentiation of human myeloid precursor cells into three different lineages and observed that a reduction in steady-state viscosity is a physiological adaptation for enhanced migration through tissues.

## 5. Active Mechanical Sorting Based on Cellular Optical Deformability

As previously discussed, cellular optical deformability, measured by OS, provides a simple marker for cells analysis, allowing to distinguish healthy, tumorigenic and metastatic cells, as well as to study the cell mechanical response from different drug treatments [8,23,63]. Starting from this evidence, the possibility to use cells’ optical deformability as a criterion for single-cell sorting was recently demonstrated, by two separate studies [24,25], thus opening the way to biological analysis requiring the selection and recovery of those cells that exhibit specific mechanical properties. The concept behind this result is quite simple: thanks to a real-time analysis of cell stretching (which can be realized by an automated computer process), the trapped and stretched cells are immediately recognized as “interesting or not”, and the presence of two separate outputs in the microfluidic structure allows sorting the trapped cell according to the stretching measurement result, without requiring any additional marker, like fluorescent stain [65], and without needing a large cell population [66,67,68,69].

### 5.1. Chip Layout for Cell Stretching and Sorting

**Figure 16:**
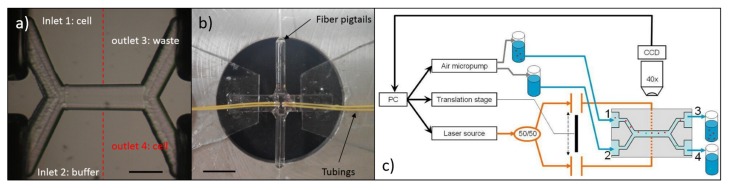
Active cell sorting chip design. (**a**) Microscope image of the internal structure of the cell sorting microchip. Scale bar: 100 µm; (**b**) The finished chip with fibers pigtailed and tubing connected is very compact. Scale bar: 1 cm; (**c**) Schematic of the experimental setup. Figure reproduced from Reference [24] with permission from Royal Society of Chemistry.

Cell sorting naturally requires more than one output, the microfluidic design has to be obviously different with respect to that of a standard OS, and also the optical section requires some modification. As an example, the optofluidic microchip proposed by Yang *et al.* [24] for active cell sorting on the basis of optical deformability is shown Figure 16 . This chip is realized in a very small piece of silica glass by the FLICE technique, as described in Section 2.4 and its size is 2 mm (thickness) × 1.5 mm (width) × 4 mm (length).

The microfluidic network design is almost the same of an optical sorter [65] and consists of an X-shaped microfluidic circuit, including two inlets, two outlets and a common central channel, see Figure 16a. The experiment setup is schematically shown inFigure 16c. After injecting cell suspension (from inlet 1) and buffer medium (from inlet 2), a laminar flow regime is built up in the central channel, allowing to keep the flowing cells in their own flow stream up to the chip outlet (port 3). Two fiber-to-fiber U benches, inserted along the optical section, provide the possibility of performing both cell stretching measurement and subsequent sorting procedure by optical forces with the same optical waveguides, by simply reducing the intensity of one beam (e.g., putting an attenuator in the corresponding U bench) and thus unbalancing the optical power levels between the two waveguides, so that the stronger beam will gently push the cell towards the desired stream flow, thus achieving cell sorting. When a cell is ready to be tested, a customized LabVIEW program is applied for the real-time analysis of optical stretching measurement; the obtained cell-deformation value is then automatically compared with a user-defined threshold, and the cell will be sorted accordingly, either to the “waste outlet” (port 3) or to the “selected cells” one (port 4). This procedure is continuously performed until the desired number of cells is sorted.

Meanwhile, another study from Faigle *et al.* [25] presented similar design but with different fabrication technique. Both the internal microfluidic channel and the optical fiber slots are created in separate glass substrate by chemical etching. The chip assembling, especially for fiber insertion and alignment, is improved by optimizing the etching and bonding geometry. The direct usage of optical fibers provides a high efficient optical power delivery from the laser source to the active stretching area and also the thin glass slide as the chip bottom layer guarantees a good imaging quality. Besides, the possibility of having more complex channel geometries can be obtained with this method.

**Figure 17:**
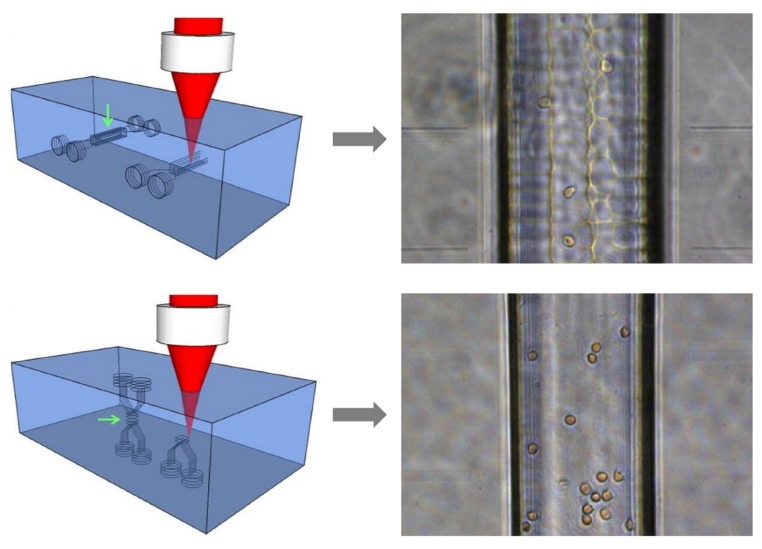
Laser writing geometry optimization for internal channel surface roughness control. Figure reproduced from Reference [24] with permission from Royal Society of Chemistry.

The microchips generally realized by the FLICE technique have the big advantage of being monolithic and compact, however, the internal surface of the microfluidic channel may be quite rough, thus causing imaging distortion and hindering imaging quality, which is fundamental for an appropriate cell contour recognition and for a correct deformation evaluation. This issue was recently solved by exploiting a new laser irradiation geometry [24], as shown in Figure 17. The main idea is to write the microchannel structure “along” the beam propagation direction, and not perpendicularly to it, as in previous fabrications. This, apparently small, change in the fabrication procedure allows defining all the microchannel surfaces in a significantly smoother way, thus strongly decreasing the internal surface roughness and greatly improving the imaging quality, as evident in Figure 17. The physical reason underlying this surface quality improvement was suggested to be connected to the highly ellipsoidal shape of the writing voxel: exploiting the newly proposed approach, all the microchannel surfaces are produced by the “longer side” of the voxel, while the “shorter and sharper” part of it has almost no impact in the structure determination, because of the voxel-translation direction.

The physical reason underlying this surface quality improvement was suggested to be connected to the highly ellipsoidal shape of the writing voxel. By using the “longer side” of the voxel and keeping the same separation between the different “writing tracks”, a larger overlap and a better uniformity of laser irradiation are therefore obtained leading to a smaller roughness after chemical etching.

### 5.2. Cell Sorting Efficiency Discussion

**Figure 18:**
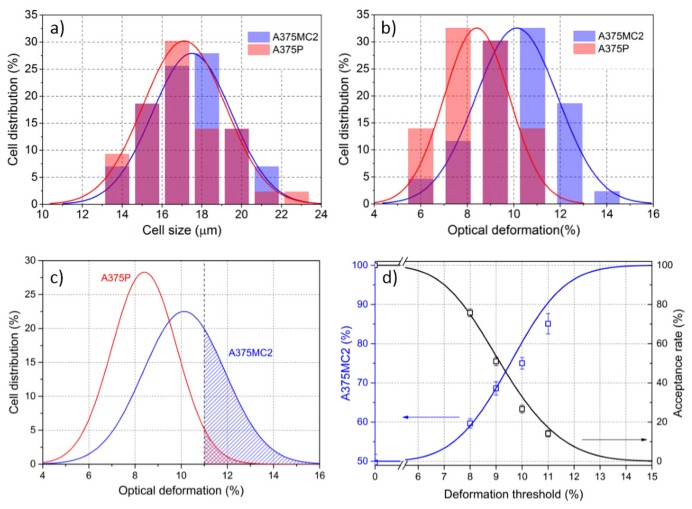
Cell sorting efficiency check. Characterization of the cellular size (**a**) and optical deformability (**b**) of two cell lines, A375MC2 and A375P; (**c**) Normalized cellular distributions as a function of their optical deformations from experiment data in (**b**). The whole area under each cell curve is set equal representing the same concentration. By defining a deformation threshold, a sub-population of A375MC2 can be enriched by collecting cells with higher deformability; (**d**) The ratio of A375MC2 in the collected cell sample and the ratio of cells in the initial sample that are expected to exhibit deformability higher than the threshold (acceptance rate) versus the defined threshold value. Figure reproduced from Reference [24] with permission from Royal Society of Chemistry.

With the new proposed microchip, Yang *et al.* performed the cell sorting experiments with metastatic (A375P) and highly metastatic (A375MC2) human melanoma cells, two cellular lines with a very similar cell size distribution (17 ± 2 µm in diameter, see Figure 18 ) and a slightly different optical deformability (8.4% ± 1.1% for A375P and 10.1% ± 1.8% for A375MC2 using two optical beams of 1.2 W each), thus reflecting their different mechanical properties and offering an intrinsic cell marker to separate them. As discussed in Section 4.2, the higher optical deformability of A375MC2 is directly related with its higher metastatic potential. In addition, it should be noted that the distributions of the cell size and optical deformability from these two cell samples follows very well a normal distribution, see the fitting Gaussian curves in Figure 18.

The cell sorting experiment was carried using a 1:1 cell mixture of A375P and A375MC2 under the same concentration (so that the same number of A375P and A375MC2 cells is present in the final suspension), thus allowing to estimate that the overall deformation distribution could be considered as the sum of the two Gaussian curves reported in Figure 18 . After each cell stretching-measurement, the produced deformation was compared with a preset threshold value, and if the measured deformation was higher than the threshold, the cell was sorted into the collection branch of outlet 4; otherwise, the cell was addressed to the waste part of outlet 3, see Figure 16. In this way, an enriched sub-population of highly metastatic cell A375MC2 can be obtained, as shown an example in Figure 18c, by selecting cell with optical deformation larger than 11%, more A375MC2 cells (blue color pattern) will be selected than A375P (red color pattern).

In order to check the efficiency of this technique, it was necessary to have a method to calculate the percentage of A374P and A375MC2 cells in the collected cell sample, and this was achieved by pre-staining the A375MC2 cells with a fluorescent dye (LDS 751). By selecting different threshold value, this cell sorting experiment was repeated and in each collected cell sample, the percentage of A375MC was calculated by simply counting the ratio of fluorescent and non-fluorescent cells. The experimental results well matched with the theoretically expected values, but a deviation form the theoretical values was obtained when a high threshold value (e.g., 11%) was used. This is connected to the fact that by increasing the threshold, the acceptance rate (*i.e.*, the number of all collected cells divided by the number of all stretched cells) is reduced, thus making the measurement longer, and giving cells the possibility to deposit on the channel or to cluster, causing undesired perturbations in the system. Similar results were also observed in the study of Faigle *et al.* [25] suggesting that the active cell sorting based on cell mechanical properties can become a reliable and useful technique for the selection of specific sub-populations of very few cells.

## 6. Optical Heating and Temperature Effect

For laser application in cell biology, heating due to the absorption of optical radiation is an important issue that should be addressed. Indeed the possible thermal damage affect both the vitality of the samples and the validity of the results.

### 6.1. Optical Heating and Temperature Measurement

In an optical tweezer, tightly focused light beams are used, causing extremely high values of light intensity (in the order of magnitude of few MW/cm^2^) to be produced in the beam focus, thus producing a significant increase of the local temperature. In particular, Peterman *et al.* [70] measured the temperature at the focus of the optical tweezer increased by 34.2 ± 0.1 K/W with 1064 nm laser for polystyrene beads of 2.2 µm diameter in glycerol medium. On the other side, OS is based on a completely different trapping configuration, obtained through two counter-propagating non-focused laser beams, as shown in Figure 1 and efficient cell trapping is achieved even using a low optical intensity from each fiber (on the order of a few mW over a large area). However, when cells-stretching measurements are conducted as described in Section 3.2, the sample flow is stopped, reducing the heat diffusion, and the trapping laser power is increased in the range between 0.5 and 1.5 W for each side, which can induce a non-negligible temperature increase.

In order to monitor the temperature change during cellular optical stretching, a precise measurement of the temperature value during the whole process should be performed. Moreover, the measurement should be done directly inside the microfluidic channel in the region illuminated by the laser radiation. However, the geometry of the micro-chip and its small dimension (100–300 µm for the internal channel) prevents the exploitation of conventional techniques as thermal sensor to perform the measurement. Additionally, it is difficult to have the spatially resolved temperature profile from the macroscopic sensor because it can only deliver an area-averaged results. All these issues were successfully overcome by a newly developed method called fluorescence ratio thermometry [71], in which laser-induced fluorescence (LIF) of two dyes, Rhodamine B and Rhodamine 110, is employed as a temperature indicator.

**Figure 19:**
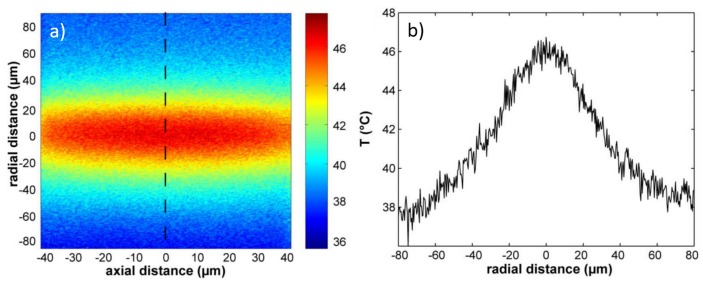
Spatial temperature profile in an optical stretcher. (**a**) Color image of the temperature increase from the two opposing laser beams in the optical stretcher. The imaging plane is the channel cross section through the center of the trap and the power of each laser beam is 1 W; (**b**) Line scan of the temperature along the dashed line in (**a**). Figure reproduced from Reference [71] with permission from Optical Society of America.

The first dye, Rhodamine B, has a well characterized temperature dependent LIF and also high temperature sensitivity (2.3% K${}^{-1}$), while the second dye, Rhodamine 110, has temperature independent fluorescence and is therefore used as a reference. The temperature is dependent on the fluorescence intensity ratio of Rhodamine B and Rhodamine 110 and the combination usage of these two dyes can avoid the problems due to the local fluctuations from the excitation light intensity or from dye concentration. The other advantage of this method is that no normalization is required and absolute temperature can be directly measured with a resolution of 2 °C. The spatial temperature profile is obtained by measuring the fluorescence intensity ratio across the channel section at the central plane of the optical trap with a laser scanning confocal microscope with spatial resolution of less than 0.5 µm. Results of the temperature distribution are shown in Figure 19a,b for a total power of 2 W (1 W per side) in the trap, highlighting a temperature rise of 25 °C over a background temperature of 21 °C. It should be noted that the temperature spatial distribution is obtained after the temperature equilibrium is established and by averaging multiple successive scan. The same temperature measurements were repeated under different laser power values from 0.5 to 1.25 W per side and the results show a linear temperature dependence on the applied laser power, with a temperature increase rate of about 13 °C/W in the laser trap center.

**Figure 20:**
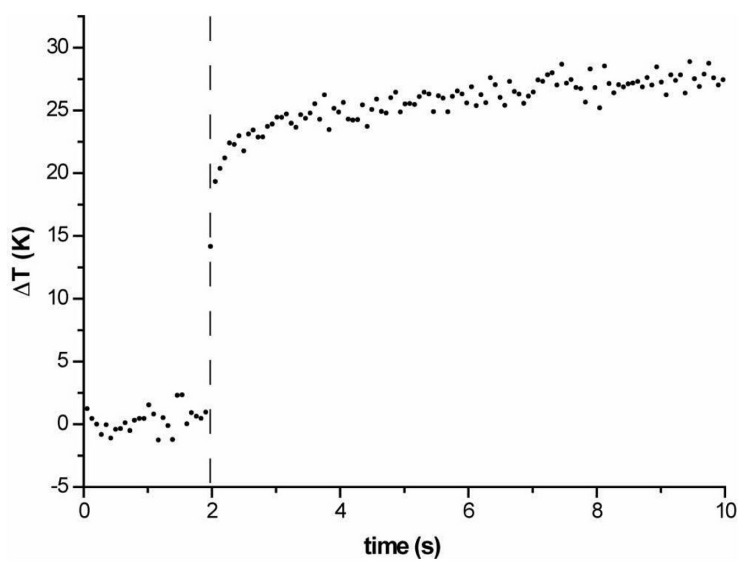
Temporal revolution of the temperature from the laser radiation in the optical stretcher. The laser is turned on at *t* = 2 s and has a total power of 2 W. Figure reproduced from Reference [71] with permission from Optical Society of America.

The same method was also applied to evaluate the temperature variation as a function of time [71,72,73], which is an important parameter, as during the stretching measurement the trapping power is abruptly increased for some seconds and is then lowered to the original trapping value, see Figure 20. In order to have a fast response and sufficient time resolution, the averaging procedure is removed and Figure 20 shows the temperature evolution for the step-like laser power increase. It can be seen that after the laser power is changed (either increased or decreased), the temperature equilibrium is simultaneously reached within fractions of a second. Wetzel *et al.* [72] also found the temperature immediately decreased to the equilibrium value after the laser is turned off.

All the temperature measurements in space and time are performed when the microfluidic flux is stopped as maintaining a medium flow would easily remove the heat from the laser irradiated region. Additionally, it should be taken into account that the temperature increase from laser heating is strongly dependent on the laser light wavelength and the cell solution medium.

Another interesting effect produced by the presence of a temperature gradient is the creation of the so called Marangoni-effect, due to a surface-tension gradient because of the temperature profile. About this point we point out that the temperature gradient is non negligible only in the direction perpendicular to the beam axis, thus allowing us to neglect its impact on the elongation in the optical beams direction. Additionally, as the cell is trapped in the position corresponding to the maximum temperature, no net force is acting on the cell, and thus no displacement from the trapping position is observed.

### 6.2. Optical Heating Impact on Cell’s Viability

As the OS is essentially used for single cell mechanics measurements, it is mandatory to investigate the optically induced heating impact on cell’s viability and properties. In particular, it must be taken into account that the cytoskeleton may be altered by cell over-stretching as well as by heating, which would result into an unaccurate mechanical characterization. Differently from the conventional research done in this field, where small temperature increases (5–10 K) and longer time spans (minutes or hours) are applied [74], during optical stretching cells experience a high temperature increase in a short time duration as discussed in Section 6.1.

Standard proliferation and epigenetic analysis are the most accurate ways to verify cellular viability. However, they normally require large number of cells and several days to fulfill the assay, which makes it not applicable if the viability of each studied cell needs to be evaluated as soon as the measurement is performed. A simple approach has been proposed in the literature [8], which suggests to observe the cell appearance through phase contrast microscopy because dead cells usually show less contrast and no clear cellular contour. Despite its easiness, this method is not fully reliable being affected by man judgment. A more careful and accurate method is the use of the vital stain: Trypan Blue allows distinguishing viable cells because the dye can penetrate only the membrane of dead or damaged cells, which can not maintain their normal functionality, while it is excluded by viable ones. Even if this method allows for single cell evaluation, it can not be applied continuously after each cell stretching measurement because cells need to be removed from the microchannel for staining and checking. A method with on-site test capability is therefore needed and can be found in a different vital stain method with calcein acetoxymethylester (AM). This dye is membrane-permeable and, more importantly, is hydrolyzed by endogenous esterases into the green fluorescent calcein, which in turn only retains in the cytoplasm of living cells [75]. By measuring and comparing the green fluorescence intensity of each cell before and after optical stretching measurement, cell viability can thus be determined. However, photo-bleaching of 10%–20% from each time imaging extremely decreases its sensitivity.

**Figure 21:**
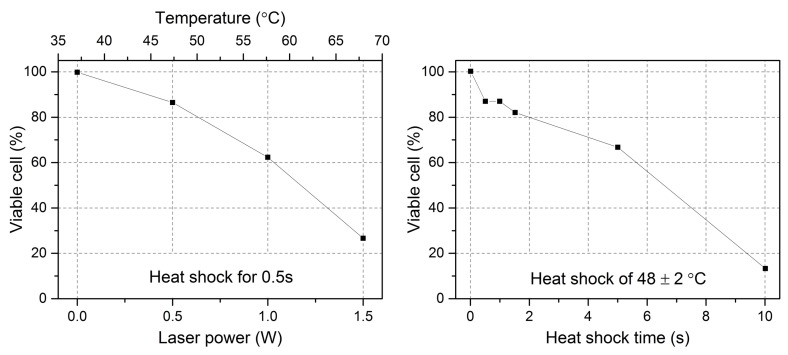
Heat shock impact on cell viability with different laser power (temperature) and time duration. The figure was realized exploiting the data reported in Reference [72].

A new method, even if with a very low throughput of less than 5 cells/h, based on cell spreading has been proposed specifically for this purpose and successfully demonstrated in literature [72]. After cell stretching measurement, the flux is not activated and the laser is completely turned off so as to let the studied cell slowly sink to the floor. After about 10 min, live cell could spread on the floor and attach to it. Since cell spreading is a vital feature of live cells, cell viability can be determined by observing cell spreading and attachment on the channel. This behavior of cell spreading involves the reassemble and rearrangement of the cell cytoskeleton and plasma and can be thus recognized as an effective indicator for cell viability. On the contrary, malignant cells show a very weak or absent spreading. By counting cells that show spreading ability, the cell viability can be easily obtained. Figure 21 shows the results of cell viability check after optical stretching in two different situations: in one case the temperature is increased by changing the laser power and in the other case by extending the stretching duration at the same laser power. It is found that more than 60% of cells can survive shorter laser heating of 0.5 s up to 58 ± 2 °C or can resist longer laser heating of 5 s at 48 ± 2 °C.

### 6.3. Temperature Effect on Cell Mechanical Property

High laser powers are needed in order to induce an appreciable cell deformation during optical stretcher measurements. Although the beams are not focused and the cells are not directly damaged by the laser radiation, they might suffer from heating due to the high laser power. Moreover, in cell optical stretching measurement, the deformation could be produced by both optical force and optical heating. In order to evaluate their contributions and better understand the temperature effect on cell mechanical property, a series of studies have been presented [39,76,77,78,79], which include variations to the standard optical stretcher to monitor and modify the temperature on the cells.

**Figure 22:**
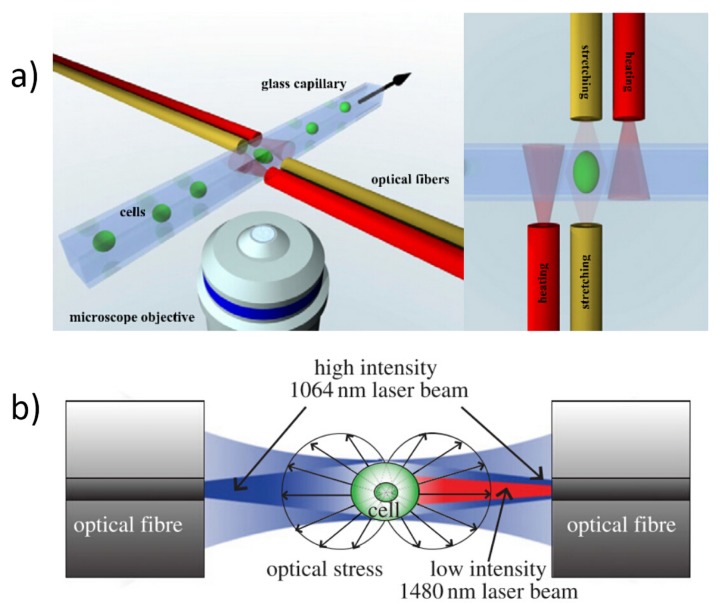
Active temperature control for optical stretcher. (**a**) Two additional fibers are added and positioned near the two opposing stretching fibers for temperature control; (**b**) Another laser is coupled into one of the two stretching fibers for temperature control. Figure reproduced from Reference [76,79] under CC-BY 3.0 license.

In Figure 22 two examples are reported where the similar idea of realizing an active temperature control is shown. The first one exploits two additional fibers, positioned beside the optical stretching ones, to introduce additional laser heating, see Figure 22a, and the same wavelength of 1064 nm is coupled in all the fibers. Since the two heating fibers are very close to the active area of cell trapping and stretching, the temperature change can be built up within one second, hence a simple temperature control for optical stretching is obtained by simply changing the power injected in the two extra fibers. The second method obtains the same effect by coupling a second laser beam into one of the two stretching fibers, see Figure 22b. The wavelength of the heating laser is chosen as 1480 nm because its absorption is higher than that of 1064 nm wavelength, leading to a strong heating at low laser power, resulting in negligible optical force from the heating laser. By changing the heating laser power, temperature increase can be easily achieved. Both methods work locally inside the microfluidic circuit and they both allow only to increase the temperature. A different temperature control system that acts on the whole microchip is obtained by mounting it in a precisely regulated temperature environment, such as, a water bath [39], an aluminum sample holder [76,77] or a thermal chamber. Differently from the previously described methods, the thermal response in these cases is much slower and they are therefore intended for long scale temperature control.

**Figure 23:**
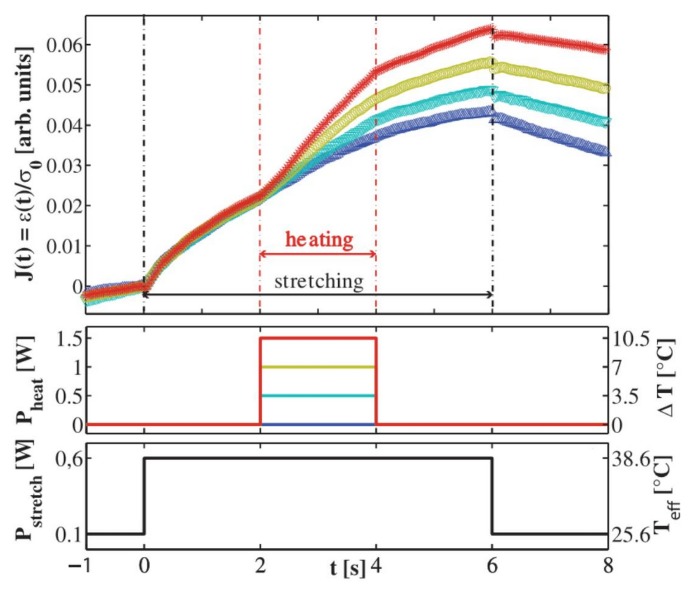
Temperature effect on cell optical deformation. Temperature is changed during the stretching measurement by using the two additional heating fibers (*P*${}_{heat}$), see Figure 22a, while keeping the stretcher power (*P*${}_{stretch}$) constant. Figure reproduced from Reference [76] under CC-BY 3.0 license.

These setups combined with an optical stretching device have been exploited to evaluate the temperature effect on cell mechanical property on different cell types, including human breast epithelial cell (MCF7, MCF10A), myeloid precursor cells (HL60) and human melanoma cells (A375MC2). Figure 23 reports an example obtained thorough the method based on extra heating-fibers, see Figure 22a. The optical compliance curves are obtained from the optical stretchering measurements both with different stretching laser power and without additional heating and with the same stretching power and different heating laser power. It can be observed that the stretching power increase leads to an strong decrease of cells’ stiffness because of their stronger deformation. By applying the additional heating radiation, cells become softer at the same stretching laser power. A thermorheological methodology based on time-temperature superposition [76] has been exploited to explain these results and to propose that the cell creep behavior from optical stretching experiment is mainly due to the temperature effect from laser heating. Under long term temperature treatment and within a certain temperature threshold, cell stretching measurements still show similar result. Consequently, temperature increase induced by laser heating from the cell optical stretching process can have a strong effect on cell mechanical properties and should be always taken into account for a proper characterization.

## 7. Other Related Studies

Additionally to the above mentioned aspects and applications of the OS, new interesting studies are carried out by combining the OS with other techniques so as to integrate new functionalities. One example exploits resonant acoustic waves to prefocus the flowing cells at the correct channel height, so that they all intercept the laser beams and are therefore suitable for laser trapping and stretching. This idea was firstly proved by Khoury *et al.* [80] in a three layer assembled optical stretcher (a thin piece of PDMS with the microfluidic channel is sandwiched between two glass slides): the acoustic wave was driven by a piezo-ceramic attached beneath the chip. However, the plastic layer, having an acoustic impedance similar to that of water, allowed only for a low-efficiency excitation of the acoustic wave, and also required a fine selection of the bottom and top glass thickness.

**Figure 24:**
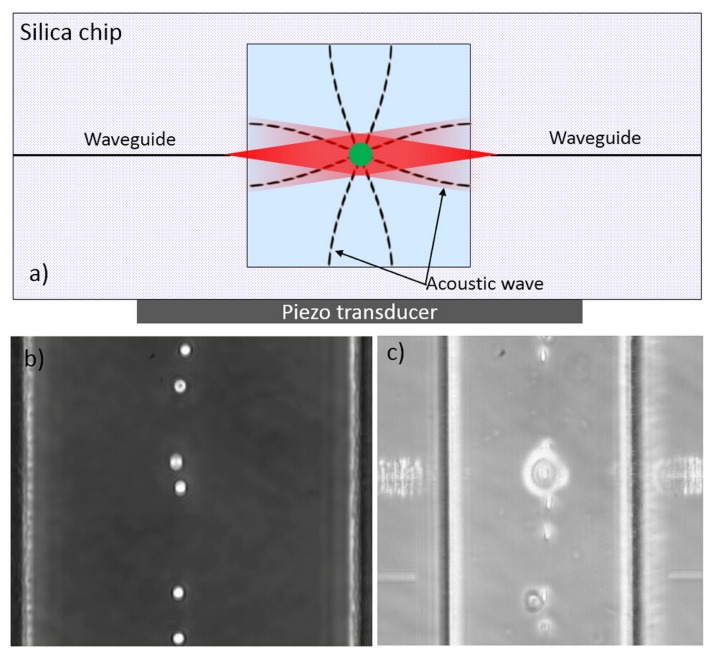
Acoustic prefocusing for optical stretcher. (**a**) Schematic illustration of the all glass microchip with both acoustic actuation (the black dash lines) driven by the underneath piezo ceramic and optical radiation (the red shaded area) emanating from the integrated waveguides. The microfluidic channel has a square cross section, 150 µm × 150 µm; (**b**) Microscope image of polystyrene beads trapped by acoustic wave in the middle of the microfluidic channel both horizontally and vertically (all beads are in the same focus); (**c**) Microscope image of red blood cells prefocused with acoustic wave for continuous optical stretching. Two opposing lasers from the waveguides are visible because of the light scattering.

A new study from Nava *et al.* recently exploited the same principle into an all-silica optical stretcher [37]. In Figure 24a the layout of the used monolithic OS is shown. Thanks to the use of a hard material, with an acoustic impedance very different from that of water, the efficiency of the acoustic wave excitation was strongly increased with respect to past result [80], and the use of a square-section channel allowed obtaining a 2D prefocusing effect (*i.e.*, acting both in vertical and horizontal direction) as shown in Figure 24b,c. The use of this chip allowed the users to greatly reduce the problems related to the height of flowing cells, thus doubling the OS measurement throughput. It was also demonstrated that the applied acoustic wave had no discernible effect on the cellular optical deformability on either red blood cells or mouse fibroblast cells. Besides, Yang *et al.* [81] further employed this chip in another recent study measuring both optical deformability and acoustic compressibility on single cells, by optical stretching and acoustophoresis experiments respectively. They found that the cancerous cell MDA-MB231 has both higher acoustic compressibility and higher optical deformability than its normal counterpart MCF7. And also, the optical deformability and acoustic compressibility are not correlated parameters. This result highlights the possibility to increase the functionalities in an optical stretcher to analyze cells in different aspects.

**Figure 25:**
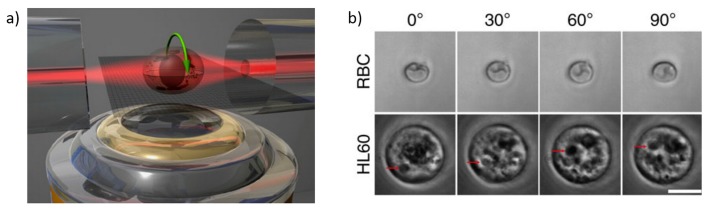
Optical cell rotator. (**a**) Illustration of the cell rotator realized in the two opposing laser radiation with an optical stretcher; (**b**) Microscope image sequences showing precise control of the cell orientation with red blood cell and HL60 cell. Figure reproduced from Reference [83] under CC-BY 4.0 license.

Another example of the integration of new functionality in optical stretcher is the optical cell rotator. Differently from the optical trapping realized with two opposing single mode fibers in an assembled optical stretcher, Kreysing *et al.* [82] replaced one of the two fibers with a dual-mode fiber and spliced it with a defined offset with respect to the original single mode fiber. By rotating this dual mode fiber, the laser beam profile is changed accordingly, leading to an active rotation of the trapped cell. With this modification, they demonstrated that individual cell can be stably held with a well defined orientation or can be rotated perpendicularly to the laser axis. Recently, Kreysing *et al.* [83] even simplified this optical cell rotator by exploiting a few mode fiber and operating it dynamically beyond the single mode regime to realize precise optical field change without physically rotating the fiber itself (see. Figure 25 ). This ability to precisely orient cells in three dimensions could enable a range of applications in biological and medical research, such as the tomographic reconstruction of cell samples, by imaging them from different angles, the determination of the 3D refractive index distributions of live cells, or wide field fluorescent imaging from multiple angles with subsequent image fusion.

## 8. Final Discussion

Since the seminal works on optical forces by Arthur Ashkin, the possibility to carefully apply controlled forces to biological elements has attracted more and more attention. The development of the optical stretcher configuration, largely investigated by Guck, Kas *et al.*, created the basis for the optimization of a new, promising and flexible tool that can be used to trap, analyze and sort single cells. In this review we highlighted the most commonly exploited fabrication technologies, we described the physical effects at the basis of the optical stretcher working mechanism, and we offered a panoramic view on some of the most relevant applications.

Currently, the main limitation of the optical stretcher is its throughput, roughly about one cell per 10 s. Even the optical stretcher is already integrated within a microfluidic circuit and the optical deformation measurement can be realized automatically in real time, this throughput is still much lower than that of flow cytometry, which is based on purely hydrodynamic cell stretching and can offer a significantly high throughput of about 1000 cells per second. In this way, the future development of optical stretcher should be focused on the feature of the precise single cell characterization and, more importantly, the subsequent analysis on the same cell with other different functionalities. Two examples have been highlighted in Section 7, where the optical cell rotator for 3D imaging or the use of acoustic waves for prefocusing in continuous optical stretching measurements and compressibility measurement have been described. In addition, for optical stretcher fabrication, an all-polymer material technique can be possible as demonstrated by a study from Khoury *et al.* [84] showing that the polymer waveguides induced by deep UV lithography [85] are integrated with microfluidic channels and fully functional for optical manipulation.

We strongly believe that the development of microfluidic systems encompassing an optical-stretcher section will largely continue in the future, and will bring to the development of off-the-shelf devices for biologists and material-science researchers, especially thanks to the possibility to include optical stretching in microfluidic devices allowing for high-resolution imaging, 3D surface tomography and integrating multiple actuators systems.
